# Multisystem injury after wasp stings in the Qinling range, Shaanxi, China: clinical profile and independent predictors of poor outcome

**DOI:** 10.3389/fmed.2025.1716614

**Published:** 2025-11-20

**Authors:** Wuyang Lv, Yaqi Ge

**Affiliations:** Department of Clinical Laboratory, Shangluo Central Hospital, Shangluo, Shaanxi, China

**Keywords:** wasp stings, multiple organ dysfunction syndrome, inflammation, metabolism, poor prognosis, independent risk factors

## Abstract

**Objective:**

This study aimed to assess the clinical characteristics and independent risk factors for adverse outcomes in multiple organ dysfunction syndrome (MODS) following wasp stings.

**Methods:**

We enrolled 187 patients admitted to the emergency department of Shangluo Central Hospital in Shaanxi Province’s Qinling area between July 2023 and November 2024 and performed a 120-day survival analysis based on follow-up records.

**Results:**

The results revealed that wasp sting-MODS involves multisystem dysfunction, characterized by altered peripheral blood cell counts, acute liver/kidney/cardiac impairment, electrolyte imbalances, and coagulation abnormalities. Inflammatory and metabolic disturbances were also prominent. Glucose and lipid metabolic disorders in these patients were closely associated with altered expression of key glucose and lipid metabolism enzymes in peripheral blood leukocytes. Among these, IL-6, TNF-*α*, IL-10, CPT1, ACO, and GLUT1 were identified as independent predictors of hospitalization exceeding 2 days, with high diagnostic efficacy. Prolonged consultation time was the only independent risk factor for adverse in-hospital outcomes. Each hour of delay increased the risk of ICU admission by 40.1%, hemodialysis requirement by 31.4%, and mortality by 20.6%. Critically, patients who sought medical care within 5 h post-sting had 8 times higher probability (95% CI: 2.3–14.5) of full recovery within 120 days after discharge compared to those with longer delays.

**Conclusion:**

Early recognition of inflammatory and metabolic biomarkers, along with medical intervention within 5 h after a wasp sting, is essential to optimize clinical management and improve outcomes in wasp sting-MODS.

## 1 Introduction

Wasps play a critical role in natural biological control and maintaining agricultural ecological balance (1). Wasp stings represent a significant public health issue, with a gradually increasing incidence rate (2). Recent extensive studies on wasp stings have revealed that their clinical manifestations vary depending on individual responses and the severity of the stings, ranging from localized hypersensitivity reactions to systemic complications such as anaphylaxis, rhabdomyolysis and multiple organ dysfunction syndrome (MODS). Severe envenomation may induce pathophysiological cascades involving mast cell degranulation, cytokine storm and direct cytotoxic effects mediated by venom components, including phospholipase A2 and hyaluronidase (3, 4). In developed countries, large-scale case reports of wasp envenomation are relatively rare. Consequently, the primary therapeutic focus following single or limited wasp stings centers on anti-allergic therapy (histamine H1/H2 receptor antagonists, corticosteroids) and desensitization protocols (1, 5). In contrast to sporadic incidents reported in developed nations, smaller-scale case studies from developing countries with distinct natural climatic and geographical conditions, mainly including China, India and South Korea, demonstrated that wasp sting may precipitate MODS. This systemic complication is associated with a progressive increase in mortality rates among victims, positioning it as an emerging public health concern (6–9).

Pathophysiological mechanisms involve direct cytotoxic effects (phospholipase A2-induced hemolysis) and immune-mediated cascades (mast cell activation and complement system dysregulation), which collectively drive organ failure in severe envenomation cases (10). Despite being recognized as a critical public health challenge, wasp sting continues to be substantially underrecognized in terms of its systemic risks, with insufficient prioritization in healthcare policies and clinical protocols. Moreover, there remains a paucity of large-cohort analyses and evidence-based reports systematically delineating the clinical profiles (organ-specific injury patterns and biomarker trajectories), therapeutic efficacy (venom immunotherapy and organ support modalities) as well as long-term prognostic determinants in patients developing wasp sting-induced MODS. This knowledge gap hinders the optimization of standardized management guidelines for severe envenomation cases (11).

China, as a large agricultural country, provides a suitable habitat for wasps because of its geographic location and natural climatic characteristics, as well as the continuing development of the return of farmland to forests. As a result, burden on public health and socio-economics (4). This is particularly evident in the northwestern and southwestern regions of China, mainly including Shaanxi, Gansu, Sichuan, and Yunnan provinces, where the incidence of MODS is significantly higher than in other regions due to the dense distribution of wasps (4, 12–14). In contrast, despite a large base of wasp stings in Europe and North American, the conversion rate of patients to MODS is low due to a well-established system of early emergency care and the determination of observation, hospitalization, admission to ICU or general ward and level of care measures based on the assessment results (15). Regarding prognostication, in Europe the poison severity score is used to assess the severity of poisoned patients (including environmental toxins) (16). In China, an Expert Consensus on Standardized Diagnosis and Treatment of Wasp Stings was released in 2018 which included guidelines for grading the severity of wasp stings but has not been widely used (12). More recently, X. Zhang et al (17) applied the Systemic Immune-Inflammatory Index (SIII) and Systemic Inflammatory Response Index (SIRI) to the assessment of wasp sting-MODS.

The objective of this study was to analyze patients with wasp sting-MODS at Shangluo Central Hospital, Shaanxi Province, from July 2023 to November 2024. Shangluo City, with a permanent resident population of 2.0412 million, is situated in southern Shaanxi Province, China, at the foothills of the Qinling Mountains. Characterized by extensive agricultural and forest coverage, the region experiences a warm-temperate semi-humid monsoon climate, with an annual average temperature of 22 °C. wasp stings is a significant clinical concern in this area, particularly during the summer-autumn season (July to November), when wasps exhibit heightened foraging activity patterns and reproductive cycles. The Qinling Mountain Range, as a hilly and mountainous terrain, provides abundant ecological niches and optimal survival conditions for wasp colonies, facilitating their proliferation.

This aim of the study is to provide novel insights into the epidemiological patterns, clinical phenotypes, independent risk determinants as well as prognostic trajectories of such cases in the Qinling Mountain region of China. This evidence-based analysis supports the development of region-specific preventive protocols, mainly including agricultural worker education on venom avoidance as well as targeted therapeutic strategies, thereby mitigating morbidity and mortality in high-risk populations.

## 2 Materials and methods

### 2.1 Ethics approval and consent to participate

This study conforms to the medical ethics standards and was approved by the Medical Ethics Committee of Shangluo Central Hospital (ethical approval number: SW2025LWLL015). The study was performed in accordance with all relevant guidelines and regulations, and informed consent was obtained from all participants and/or their legal guardians. No information or images have been used that could potentially identify study participants.

### 2.2 Study design and participants

**Figure 1:**
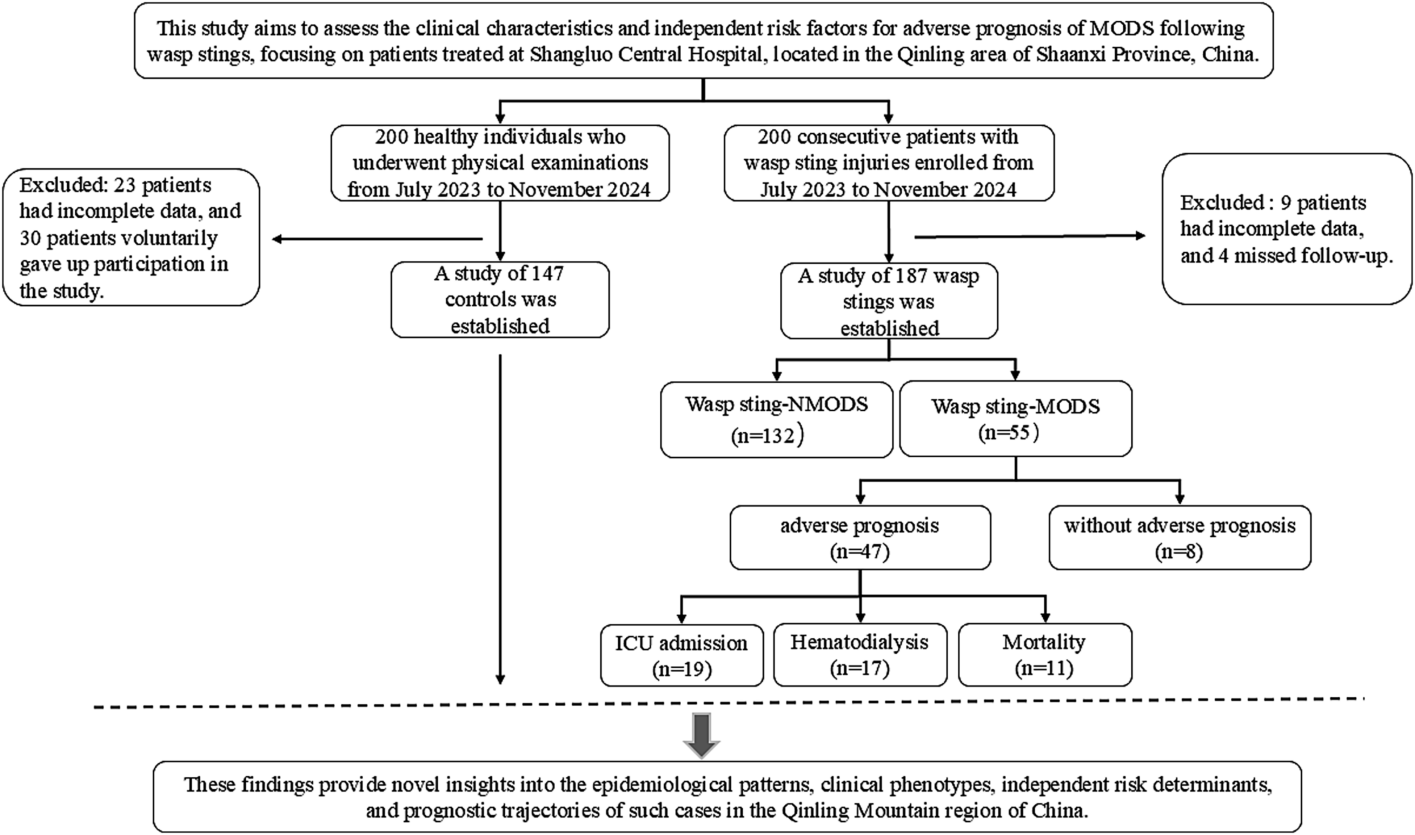
Flow chart for patient classification. MODS, multiple organ dysfunction syndrome.

This study enrolled 187 patients with wasp sting admitted to the emergency department of Shangluo Central Hospital from July 2023 to November 2024 as the research group, and selected 147 healthy individuals undergoing physical examination during the same period as the control group. Within 48 h of admission for wasp sting patients, they were further divided into Wasp Sting with non-multiple organ dysfunction syndrome group (wasp sting-NMODS) and Wasp Sting with multiple organ dysfunction syndrome (wasp sting-MODS) group, based on disease progression, inclusion criteria, and diagnostic criteria (Figure 1).

Inclusion criteria: Age ≥ 18 years; Definite history of wasp sting with typical clinical symptoms; Time from sting to presentation <48 h; Complete medical records. The exclusion criteria were as follows: Concomitant poisoning with other toxins; Presence of severe cardiovascular or cerebrovascular diseases, chronic liver or kidney dysfunction, diabetes, or immune system disorders; Presence of hemophagocytic lymphohistiocytosis (HLH) or suspected HLH based on clinical manifestations (refractory fever, hepatosplenomegaly), hematological abnormalities (unexplained pancytopenia), and bone marrow examination results (absence of hemophagocytosis); Other conditions, such as drug poisoning, trauma, strenuous exercise, or excessive alcohol consumption; patients who were using glucocorticoids or other acute infections before being stung; unable to confirm whether it was a sting from another insect; pregnancy; incomplete data collection.

In this study, we strictly adopted the 2016 Revised International Sepsis and Septic Shock Management Guidelines (SSC Guidelines) to diagnose MODS (18). This guideline is widely recognized in the international clinical and research community for its high authority and clinical applicability, ensuring the consistency of our diagnostic standards with mainstream global research. The diagnosis of MODS was determined by confirming the functional impairment of two or more major organ systems. In addition, to ensure diagnostic accuracy, we implemented the following steps, two independent attending physicians (with more than 5 years of clinical experience in emergency medicine) separately evaluated each patient’s organ function indicators.

### 2.3 Detection of research indicators and collection of clinical data

We collected demographic characteristics, comorbidities, and research test indicators for study subjects. Demographic characteristics included gender, age, blood pressure, history of underlying diseases, family genetic history, and surgical history. Research test indicators were categorized into three main types: complete blood count (CBC), biochemical profile, and coagulation function tests. Among them, the indicators for CBC include: White blood cell (WBC), Red blood cell (RBC), Hemoglobin (HGB), Platelet (PLT); The indicators for biochemical profile include: Alanine transaminase (ALT), Glutamyltransferase (GGT), Alkaline phosphatase (ALP), Adenosine deaminase (ADA), Monoamine oxidase (MAO), A-L-fucosidase (AFU), 5-nucleotidase (5’-NT), Creatinine (Cr), Blood urea nitrogen (BUN), Cystatin C (Cys-c), Creatine kinase (CK), Creatine kinase-MB (CK-MB), Lactic dehydrogenase (LDH), High sensitive troponin I (TNHS), Myoglobin (Mb), Kalium (K), Natrium (Na), Chlorine (CL), Ionic Calcium (nCa); The indicators for coagulation function include: Prothrombin Time (PT), Activated partial thromboplastin time (APTT), Thrombin time (TT), Fibrinogen (FIB), D-Dimer (D-D), Fibrinogen degradation products (FDP). Additionally, the length of hospitalization and clinical outcomes (survival, mortality, or complications) of patients in the Wasp sting-MODS group were systematically documented.

### 2.4 Specimen collection and processing protocols

Venous blood samples were collected from enrolled subjects within 48 h post-admission. For each patient, three venous blood samples were obtained and allocated to the following analyses: CBC (2 mL of venous blood was collected in EDTA-K2 anticoagulant vacutainer tubes, gently inverted 8–10 times to ensure proper mixing, and analyzed within 10 min of collection using standardized protocols), Serum biochemical analysis (4 mL of venous blood was collected in coagulant activator tubes, clotted at room temperature for 30 min, and centrifuged at 3500 × g for 10 min at room temperature to isolate serum. The separated serum was analyzed within 30 min post-separation using standardized laboratory protocols), and Coagulation profile (3 mL of venous blood was collected in sodium citrate anticoagulant tubes, gently inverted 8–10 times to ensure homogeneous mixing, and centrifuged at 3500 × g for 15 min at room temperature to obtain plasma. The separated plasma was analyzed within 30 min post-processing using standardized laboratory protocols).

Hematological parameters were analyzed using an automated hematology analyzer (Sysmex Corporation, XN-9000, Japan), serum biochemical parameters were measured with an automated biochemistry analyzer (Beckman Coulter AU5800, United States), and coagulation profiles were assessed via an automated coagulation analyzer (Werfen, ACLTOP750, China). Subsequently, the remaining serum after the test was aliquot in sterile EP tubes and stored at −80 °C for subsequent research.

### 2.5 Detection of inflammation and metabolic indicators

To observe the expression of inflammatory factors and metabolic indicators in serum of patients in the different groups. Serum samples were taken from −80 °C and thawed slowly on ice. Next, serum was centrifuged at 400 g for 30 s and assayed in strict accordance with the operation manual. The expression of interleukin-1 (IL-1), IL-6, tumor necrosis factor-alpha (TNF-*α*) and IL-10 in serum were determined by human ELISA kits (Jianglai Biology, Shanghai, China), respectively. On the other hand, the levels of metabolic indicators in serum, mainly including total cholesterol (TC), triglycerides (TG), low-density lipoprotein (LDL), high-density lipoprotein (HDL), lactic acid (LAC), glucose (GLU) and C-reactive protein (CRP), were all detected and obtained by the automated biochemistry analyzer.

### 2.6 Isolation and collection of human peripheral blood leukocytes

2 mL of fresh anticoagulated whole blood was transferred into a sterile centrifuge tube, centrifuged at 400–500 × g for 5 min at room temperature, and the supernatant was carefully aspirated and discarded. 7 mL of erythrocyte lysis buffer (Beyotime, C3702, China) was added to the cellular pellet, gently pipetted to homogenize, and incubated at room temperature for 5 min with intermittent gentle agitation to facilitate erythrocyte lysis. Subsequently, the mixture was centrifuged at 500 × g for 5 min at 4 °C, and the reddish supernatant (containing lysed erythrocyte debris) was aspirated and discarded. If incomplete erythrocyte lysis was observed, two additional rounds of the aforementioned procedure were performed to ensure complete removal of residual erythrocytes, five volumes of ice-cold sterile phosphate-buffered saline (Solarbio, P1003, China) was added to the cellular pellet, which was then resuspended thoroughly before centrifugation at 500 × g for 3 min at 4C. Following aspiration and removal of the supernatant, the washing procedure was repeated twice to remove residual debris and the human peripheral blood leukocyte pellet was obtained for downstream applications. Finally, the leukocyte pellet was resuspended in 50 μL of Diethylpyrocarbonate-treated water (Beyotime, R0021, China) as required by the experimental protocol, transferred to a sterile microcentrifuge tube, and stored at −80 °C for subsequent experimental analyses.

### 2.7 RNA extraction and RT-qPCR

Gene expressions of human peripheral blood leukocytes were determined by RT-qPCR. Briefly, total RNA was isolated from human peripheral blood leukocytes using RNAiso Plus reagent (Code No.9109, Takara) according to the manufacturer’s protocol. RNA was reverse-transcribed (RT) to cDNA by using a Script cDNA Synthesis Kit (Code No. RR036A, Takara). Quantitative RT-PCR was performed using the Bio-Rad CFX96 Optics Real-Time PCR System (Bio-Rad Laboratories, Inc., CA, United States). The following PCR conditions were used for purpose gene: 40 cycles of denaturation at 95 °C for 5 s, annealing at 60 °C for 30 s, and extension at 72 °C for 15 s. The primer sequence was listed in Table 1.

**Table 1:** The primer sequences.

Genes	Forward primer (5′-3′)	Reverse primer (5′-3′)
GLUT1	GATGAAGGAAGAGAGTCGGCAGATG	CAGCACCACAGGCGATGAGGATG
GK	GACAAGGGCATCCTCCTCAATTGGA	CTAGACAAGGGCATCCTCCTCAATT
LDH	ATCTTGACCTACGTGGCTTGGA	CCATACAGGCACACTGGAATCTC
PDK1	ACCAGGACAGCCAATACAAG	CCTCGGTCACTCATCTTCAC
PKM2	ATTATTTGAGGAACTCCGCCGCCT	ATTCCGGGTCACAGCAATGATGG
ACC	TGGATTTTTTGATTATGGCTCTTTC	CCTGGCTCTGCCAACTACCA
FAS	CAGGAACAACTCATCCGTTCTCT	GGACCGAGTAATGCCGTTCA
ACO	CTTCGTGCAGCCAGATTG	CTACTTCCTTGCTCTTCCTGTGACT
CPT1	CCTTGGCTACTTGGTACGAAT TCT	GCGGATGCAGTGGGACAT
FABPpm	TCTGCTTCACCGGCCTAAA	AGATTCGACCATCCTTTGTCATG
SREBP1c	CAGGTCCTTGAGCTCCACAAT C	GCCCACAATGCCATTGAGA

GLUT1, Glucose transporter protein 1; GK, glucokinase; LDH, lactate dehydrogenase; PDK1, pyruvate dehydrogenase kinase; PKM2, pyruvate kinase M2; ACC, acetyl-CoA carboxylase; FAS, fatty acid synthase; ACO, acyl-CoA oxidase; CPT1, carnitine palmitoyl-transferase; FABPpm, fatty acid binding protein; SREBP1c, sterol regulatory element binding protein 1c.

### 2.8 Statistical analysis

The data were analyzed using SPSS 22.0 statistical software. Normally distributed continuous variables were expressed as mean ± standard deviation (x̄ ± SD). Inter-group comparisons were performed using the independent samples t-test. Non-normally distributed continuous variables were reported as median (*P_25_*, *P_75_*). Inter-group comparisons were conducted using the Mann–Whitney U test or Fisher’s exact test Categorical data were described using frequency and percentage. Inter-group comparisons were analyzed with the chi-square test (χ^2^ test). Univariate logistic regression models were employed to analyze the risk factors (inflammatory and metabolic indicators) associated with hospitalization duration >2 days in patients with wasp sting-induced MODS. Variables with *p* < 0.01 in the univariate analysis were incorporated into a multivariate logistic regression model, combined with multiple linear regression analysis, to identify independent risk factors influencing hospitalization duration. A receiver operating characteristic curve (ROC) was plotted to evaluate the predictive value and diagnostic efficacy of these independent risk factors for prolonged hospitalization (>2 days). Furthermore, a logistic regression model combined with random forest analysis was utilized to identify independent risk factors for adverse outcomes (ICU admission, hemodialysis, and mortality) during hospitalization in patients with wasp sting-induced MODS. The methodology involved univariate screening of variables, followed by multivariate logistic regression analysis, and subsequent integration with random forest modeling to refine risk factor selection. Survival analysis was further performed to evaluate the predictive value of these independent risk factors for complete recovery (local symptoms and systemic symptoms disappear, the function of the damaged organs recovers to the level before the wasp sting, and there are no long-term sequelae or functional disorders) within 120 days post-discharge. A *p*-value <0.05 was considered statistically significant.

## 3 Results

### 3.1 General information and clinical manifestations

As shown in Table 2, in baseline information, the wasp sting-MODS group exhibited the highest proportion of females and the oldest mean age, but these differences across the three groups were not statistically significant (*p* > 0.05). Notably, the prevalence of comorbidities in the wasp sting-MODS group, primarily hypertension and diabetes, was significantly higher compared to the other two groups (*p* < 0.05; Table 2).

**Table 2:** Baseline characteristics of the study population.

Baseline characteristics	Control (*n* = 147)	Wasp sting-NMODS (*n* = 132)	Wasp sting-MODS (*n* = 55)	*p* value
Gender				
Male	81(55.1%)	65(49.2%)	22(40%)	0.153
Female	66(44.9%)	67(50.8%)	33(60%)	
Age (years)	55.09 ± 12.12	57.64 ± 14.55	59.18 ± 8.17	0.074
Hypertension (%)	19(12.9%)	14(10.6%)	14(25.5%)	0.025
Diabetes (%)	5(3.4%)	9(6.8%)	8(14.5%)	0.017
Family genetic history (%)	3(2.0%)	6(4.5%)	3(5.4%)	0.383
Surgical history (%)	12(8.2%)	14(10.6%)	5(9.1%)	0.780
Number of stings				
N ≥10	0	11(8.3%)	6(10.9%)	0.435
5 < N < 10	0	37(25.2%)	15(27.3%)	0.761
N < 5	0	84(63.6%)	29(61.8%)	0.165
Time of consultation (hours)	0	4.30 ± 0.61	12.65 ± 2.76	<0.0001
Hemolysis (%)	0	5(3.8%)	40(72.7%)	<0.0001
Hematuresis (%)	0	2(1.5%)	34(61.8%)	<0.0001
Acute kidney injury (%)	0	0	55(100%)	-
Acute hepatic injury (%)	0	0	55(100%)	-
Myocardial damage (%)	0	0	55(100%)	-
Duration of hospitalization (day)	0	1.69 ± 0.46	13.04 ± 3.27	<0.0001
ICU (%)	0	0	19(34.5%)	-
Hematodialysis (%)	0	0	17(30.9%)	-
Mortality (%)	0	0	11(20.0%)	-

MODS, Multiple organ dysfunction syndrome; NMODS, Non-multiple organ dysfunction syndrome; ICU, Intensive Care Unit.

In terms of clinical characteristics, the proportion of wasp sting-MODS group with ≥ 10 stings was 10.9%, which was not statistically different from the wasp stings-NMODS and control group (*p* > 0.05). Notably, the time to consultation was significantly delayed in the wasp sting-MODS group, which was significantly higher than in the wasp sting-NMODS and control group (*p* < 0.0001; Table 2).

Crucially, among the clinical outcomes, the incidence of hemolysis, hematuria, AKI, acute hepatic injury, and myocardial injury was significantly higher in the wasp sting-MODS group compared to the other two groups (Table 2). The length of hospitalization was prolonged in the wasp sting-MODS group, which was significantly higher than in the wasp sting-NMODS and control group (*p* < 0.0001; Table 2). In addition, ICU, hemodialysis and mortality rates in the wasp sting-MODS group reached 34.5, 30.9 and 20.0%, respectively (Table 2).

These findings demonstrated that patients with wasp sting-MODS exhibited more severe disease progression and poorer prognoses, characterized by a higher prevalence of comorbidities, delayed consultation time, elevated incidence of hemolysis and hematuria, and higher rates of AKI, hepatic injury and myocardial injury, as well as prolonged hospitalization and increased mortality rates.

### 3.2 Multisystem abnormalities and systemic pathophysiological alterations in wasp sting-MODS

This study conducted comprehensive analyses of peripheral blood parameters, liver/renal/cardiac function, electrolyte balance and coagulation in patients with wasp sting-MODS. The results demonstrated that patients with wasp sting-MODS exhibited significantly elevated leukocyte series indices, markedly reduced erythrocyte and platelet series indices in peripheral blood compared to the wasp sting-NMODS and control group (Table 3; Supplementary Table 1). (ALT, AST, GGT, ALP, ADA, MAO, AFU, 5’-NT) was higher than those in the other two groups, suggesting that liver function was seriously damaged (Table 3; Supplementary Table 1). Similarly, (Cr, BUN, UA, Cys-c, CK, CK-MB, LDH, HBDH, TNHS, Mb) were significantly increased in the wasp sting-MODS group compared with the other two groups, suggesting acute injury to renal/cardiac function (Table 3; Supplementary Table 1). Simultaneously, the wasp sting-MODS group exhibited significantly elevated (K, nCa) and markedly reduced (Na, CL, Mg, P) levels compared to the wasp sting-NMODS and control group, indicating severe electrolyte disturbances (Table 3; Supplementary Table 1). Additionally, the coagulation homeostasis was disrupted in patients with wasp sting-MODS, which was mainly characterized by a prolonged clotting time (elevated APTT, PT and TT), a dramatically reduced FIB, and a significantly elevated expression of D-D and FDP (Table 3).

**Table 3:** Comparison of laboratory data of controls, wasp sting-NMODS and wasp sting-MODS group.

Laboratory data	Control (*n* = 147)	Wasp sting-NMODS (*n* = 132)	Wasp sting-MODS (*n* = 55)	*p* value
Peripheral blood				
WBC(×10^9^/L)	5.99 ± 1.81	12.76 ± 6.04	17.50 ± 7.34	<0.0001
RBC(×10^12^/L)	4.77 ± 0.50	4.45 ± 0.52	3.72 ± 0.82	<0.0001
HGB(g/L)	144.74 ± 15.61	137.50 ± 18.7	118.58 ± 25.46	<0.0001
PLT(×10^9^/L)	219.0(187.0, 268.0)	233.0(181.0, 274.0)	132.0(96.0, 178.0)	<0.0001
Liver				
ALT(U/L)	24.29 ± 12.82	23.48 ± 9.96	549.37 ± 195.29	<0.0001
GGT(U/L)	23.26 ± 7.56	21.95 ± 12.05	352.64 ± 107.22	<0.0001
ALP(U/L)	90.88 ± 26.14	90.46 ± 26.87	399.60 ± 136.51	<0.0001
ADA(U/L)	9.21 ± 3.38	12.13 ± 2.82	167.04 ± 58.01	<0.0001
MAO(U/L)	6.47 ± 3.15	8.99 ± 3.65	118.23 ± 27.26	<0.0001
AFU(U/L)	25.32 ± 5.83	24.15 ± 6.64	212.14 ± 59.50	<0.0001
5’-NT(U/L)	6.68 ± 1.96	7.64 ± 3.54	169.73 ± 58.24	<0.0001
Kidney				
Cr(μmol/L)	68.62 ± 15.16	66.86 ± 19.92	253.82 ± 82.86	<0.0001
BUN(mmol/L)	4.39 ± 1.27	6.17 ± 2.17	16.28 ± 5.21	<0.0001
Cys-c(mg/L)	0.97 ± 0.17	0.88 ± 0.17	10.04 ± 3.11	<0.0001
Myocardium				
CK(U/L)	82.0(67.0, 125.0)	155.0(124.0, 185.3)	999.0(636.5, 1343.5)	<0.0001
CK-MB(U/L)	15.0(11.0, 20.0)	20.0(15.0, 25.8)	225.0(160.5, 259.0)	<0.0001
LDH(U/L)	182.0(157.0, 214.0)	224.5(199.3, 250.0)	801.0(572.5, 1212.0)	<0.0001
TNHS(pg/ml)	9.6(5.8, 13.4)	5.4(2.8, 10.9)	956.3(813.5, 1115.4)	<0.0001
Mb(μg/ml)	47.0(30.0, 62.0)	58.3(40.5, 73.9)	1984.4(1256.7, 2404.3)	<0.0001
Electrolyte				
K(mmol/L)	4.33 ± 0.48	3.77 ± 0.48	5.76 ± 0.76	<0.0001
Na(mmol/L)	142.18 ± 3.25	140.04 ± 3.07	135.87 ± 3.46	<0.0001
CL(mmol/L)	104.97 ± 3.23	103.41 ± 3.21	98.76 ± 3.95	<0.0001
nCa(mmol/L)	1.40 ± 0.30	1.23 ± 0.26	2.02 ± 0.43	<0.0001
Coagulation				
PT(s)	12.45 ± 1.19	12.31 ± 3.81	32.40 ± 9.13	<0.0001
APTT(s)	29.3(27.0, 31.6)	30.0(25.0, 41.5)	118.3(57.4, 128.6)	<0.0001
TT(s)	19.86 ± 2.84	17.83 ± 4.16	97.82 ± 22.95	<0.0001
FIB(g/L)	2.9(2.6, 3.5)	2.9(2.3, 3.4)	1.1(0.8, 1.4)	<0.0001
D-D(mg/L)	0.28(0.21, 0.39)	1.36(0.42, 4.14)	25.0(17.3, 32.1)	<0.0001
FDP(μg/mL)	2.7(1.8, 3.7)	4.5(2.3, 8.5)	25.4(20.3, 33.4)	<0.0001

WBC, White blood cell; RBC, Red blood cell; HGB, Hemoglobin; PLT, Platelet; the indicators for biochemical profile include: ALT, Alanine transaminase; GGT, Glutamyltransferase; ALP, Alkaline phosphatase; ADA, Adenosine deaminase; MAO, Monoamine oxidase; AFU, A-L-fucosidase; 5’-NT, 5-nucleotidase; Cr, Creatinine; BUN, Blood urea nitrogen; Cys-c, Cystatin C; CK, Creatine kinase; CK-MB, Creatine kinase-MB; LDH, Lactic dehydrogenase; TNHS, High sensitive troponin I; Mb, Myoglobin; K, Kalium; Na, Natrium; CL, Chlorine; nCa, Ionic Calcium; the indicators for coagulation function include: PT, Prothrombin Time; APTT, Activated partial thromboplastin time; TT, Thrombin time; FIB, Fibrinogen; D-D, D-Dimer; FDP, Fibrinogen degradation products.

In summary, the wasp sting-MODS group showed significant abnormalities in several aspects such as peripheral blood parameters, liver/kidney/cardiac function, electrolyte balance and coagulation function, which were mainly manifested as acute damage to liver, kidney and cardiac functions as well as electrolyte and coagulation disorders, suggesting that wasp sting-MODS group has serious systemic effects.

### 3.3 Characteristics of metabolic disturbances and inflammatory imbalances in wasp sting-MODS

In previous results, we observed significant changes in laboratory data in patients with wasp sting-MODS group compared to the wasp sting-NMODS and control group. Next, to further explore changes in inflammatory and metabolic markers, the serum levels of glucose, lipids and the inflammatory factors in three groups were analyzed.

**Figure 2:**
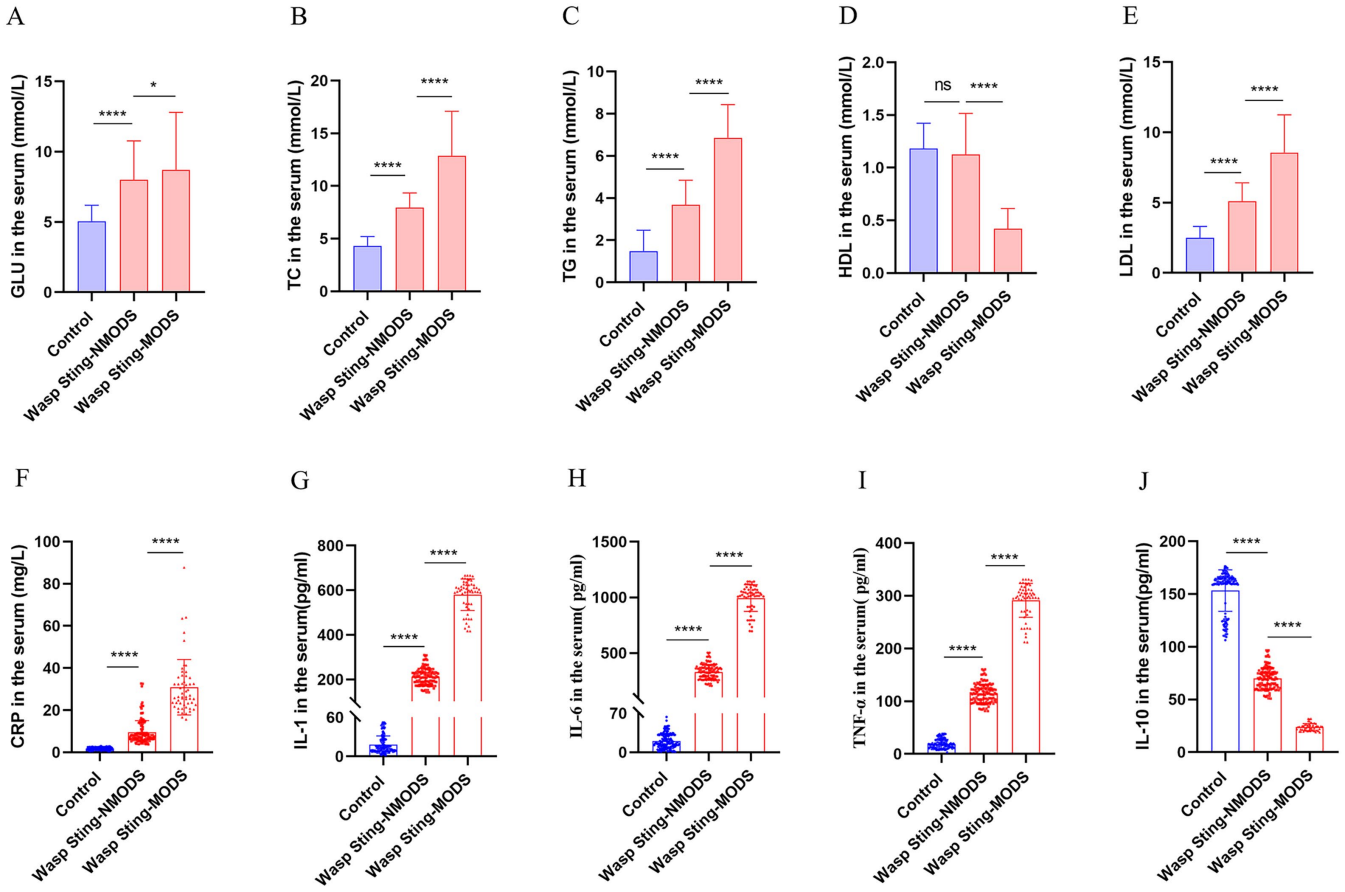
The inflammatory and metabolic expressions of patients in different groups. **(A)** The level of GLU in the serum. **(B–E)** The expression of blood lipids in serum, mainly including TC, TG, LDL and HDL. **(F–J)** Inflammatory indicators in serum, mainly manifested as CRP, IL-1, IL-6, TNF-*α* and IL-10. *p < 0.05, **p < 0.01, ***p < 0.001, ****p < 0.0001, or ns *p* > 0.05.

**Figure 3:**
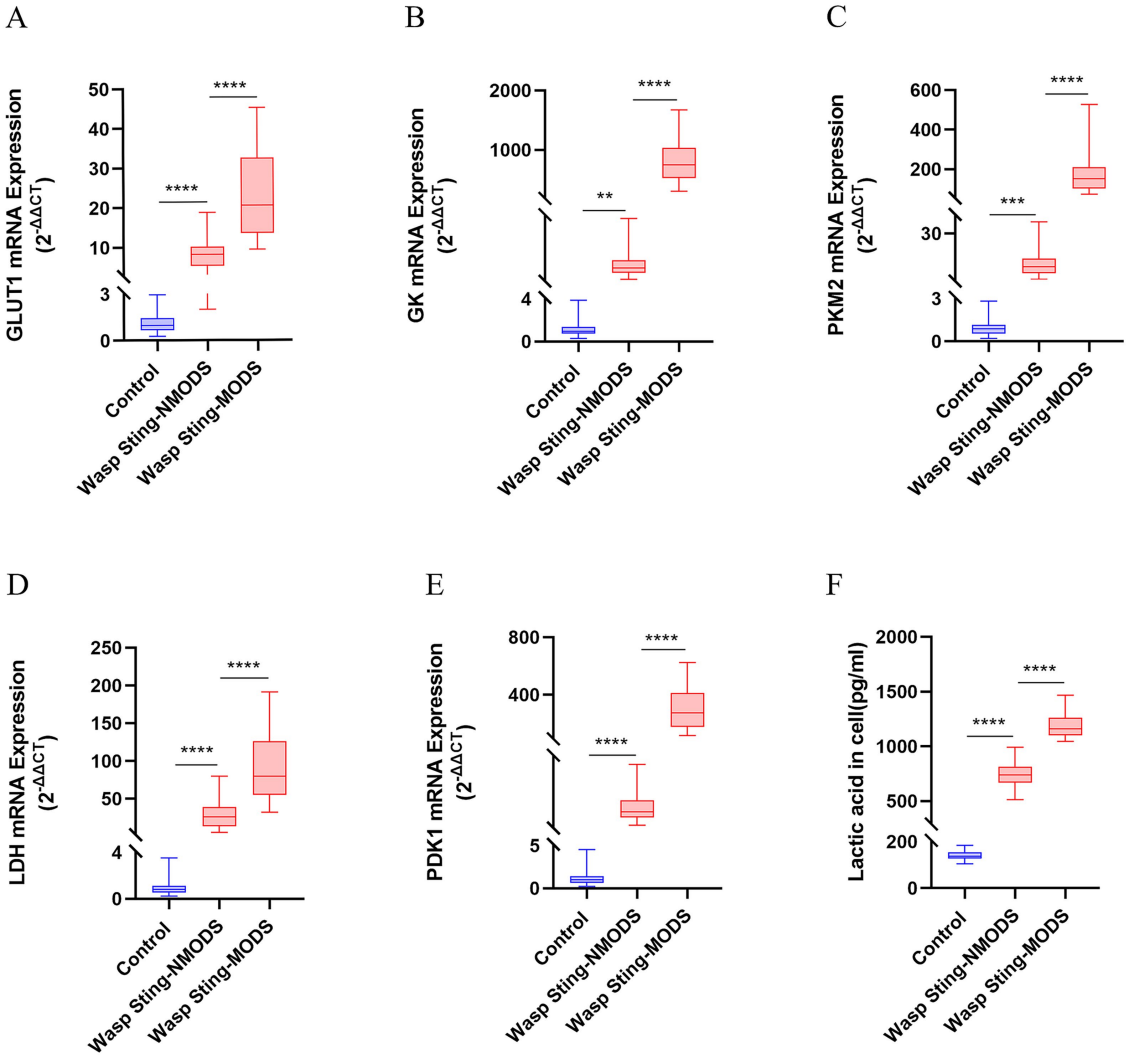
The expression of key enzyme genes for glucose metabolism. **(A–E)** The mRNA expressions of GLUT1, GK, PKM2, LDH and PDK1 in the peripheral blood leukocytes of patients in different groups were detected by RT-qPCR. **(F)** The level of LAC in the serum was evaluated. *p < 0.05, **p < 0.01, ***p < 0.001, ****p < 0.0001, or ns *p* > 0.05.

The results revealed that, in the spectrum of metabolic disturbances, the wasp sting-MODS group exhibited a progressive elevation in serum GLU, LAC, TC, TG and LDL levels with disease progression, alongside a gradual decline in HDL levels compared to the wasp sting-NMODS and control group (Figures 2A–E, 3F). In terms of inflammatory cytokines, the wasp sting-MODS group exhibited significantly elevated levels of pro-inflammatory mediators, including CRP, IL-6, IL-1 and TNF-*α* compared to the wasp sting-NMODS and control group (Figures 2F–I). Conversely, the anti-inflammatory cytokine IL-10 was markedly reduced in the wasp sting-MODS group (Figure 2J), indicating impaired anti-inflammatory compensatory capacity and a shift toward systemic hyperinflammation.

In summary, the wasp sting-MODS group demonstrated a distinct pattern of progressive metabolic dysregulation and a significant systemic hyperinflammation.

### 3.4 The gene expression of key enzymes involved in glucose and lipid metabolism were significantly upregulated within peripheral blood leukocytes in wasp sting-MODS

**Figure 4:**
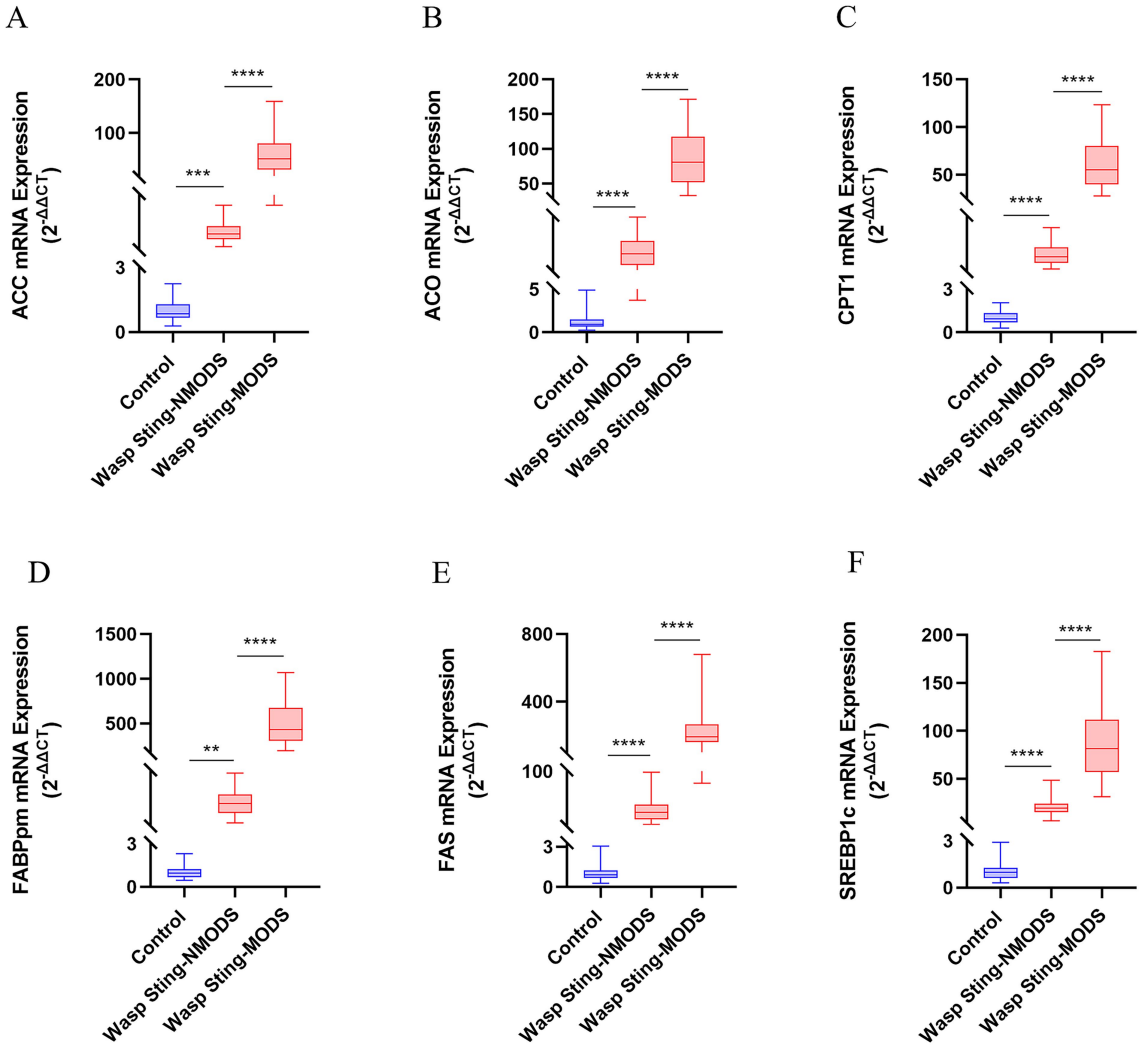
The expression of key enzyme genes for lipid metabolism. **(A–F)** The mRNA expressions of ACC, ACO, CPT1, FABPpm, FAS and SREBP1c in the peripheral blood leukocytes of patients in different groups were detected by RT-qPCR. *p < 0.05, **p < 0.01, ***p < 0.001, ****p < 0.0001, or ns *p* > 0.05.

Preliminary experiments showed that peripheral blood leukocyte counts were significantly higher in the wasp sting-MODS group. Based on this finding, we hypothesized that the co-occurrence of metabolic disorders and inflammatory imbalances in patients with wasp sting-MODS may be related to reprogramming of glycolipid metabolism in peripheral blood leukocytes. To validate this hypothesis, we analyzed the gene expression of key enzymes in glucose and lipid metabolism in peripheral blood leukocytes from patients using RT-qPCR. The results demonstrated that the wasp sting-MODS group exhibited a progressive and significant upregulation of gene expression for glucose metabolism enzymes (GLUT1, GK, PKM2, LDH, PDK1) and lipid metabolism enzymes (ACC, ACO, CPT1, FABPpm, FAS, SREBP1c) compared to the wasp sting-NMODS and controls group, which correlated with worsening disease severity (Figures 3A–E, 4A–F). This finding bridges the initial observation of leukocytosis to the investigation of leukocyte glycolipid metabolic reprogramming, suggesting a potential association between the two.

### 3.5 Assessment of prognostic value and diagnostic efficacy of inflammatory imbalance and metabolic dysregulation indices in predicting prolonged hospitalization (>2 days) in the wasp sting-MODS

**Figure 5:**
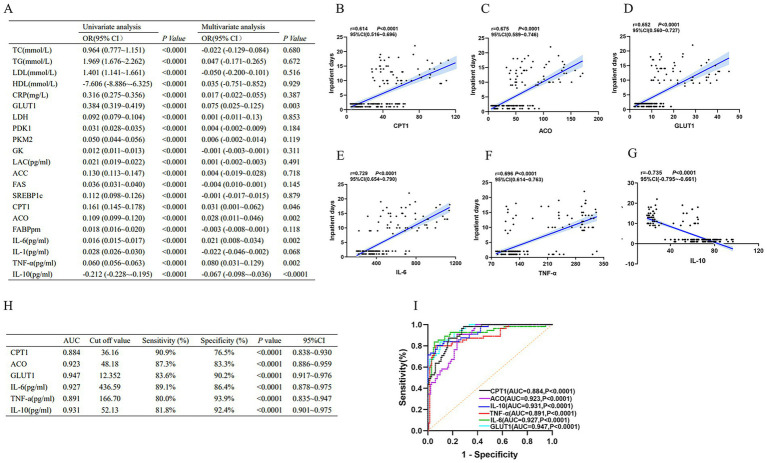
Biomarkers of inflammatory imbalance and metabolic dysregulation in patients with wasp sting-MODS. **(A)** A univariate combined multifactorial logistic approach screened six independent predictors, namely IL-6, TNF-α, IL-10, CPT1, ACO and GLUT1. **(B–G)** Among the six independent predictors screened, IL-6, TNF-α, IL-10, CPT1, ACO, and GLUT1 were each significantly and positively associated with days of hospitalization, whereas IL-10 was negatively associated with days of hospitalization. **(H,I)** Evaluation by ROC curve showed high diagnostic efficacy for IL-6, TNF-α, IL-10, CPT1, ACO and GLUT1. *p < 0.05, **p < 0.01, ***p < 0.001, ****p < 0.0001, or ns *p* > 0.05.

Firstly, we assessed the predictive value of indicators of inflammatory imbalance and metabolic disorders in patients with wasp stings-MODS who were hospitalized for more than 2 days. As shown in Figure 5A, in the results of univariate factor analysis, elevated pro-inflammatory factors and dyslipidemia were significantly and positively associated with hospitalization duration, whereas the anti-inflammatory factors IL-10 and HDL were significantly and negatively associated with hospitalization duration, suggesting that both enhanced inflammatory response and dyslipidemia (especially high TG, LDL and low HDL) may extend the duration of hospitalization. In addition, key enzyme indices of glucose metabolism and lipid metabolism were both significantly and positively correlated with hospitalization days, indicating that metabolic disorders are also associated with prolonged hospitalization duration. Next, we further adjusted for confounders by multivariate analysis, which showed that CPT1, ACO, GLUT1, IL-6, TNF-*α* and IL-10 were all independent predictors of prolonged hospitalization (Figure 5A). Among them, CPT1, ACO, GLUT1, IL-6, and TNF-*α* expression were significantly positively correlated with duration of hospitalization, while IL-10 expression was significantly negatively correlated with duration of hospitalization (Figures 5A–G). These indicators may serve as important biomarkers for predicting prolonged hospitalization (>2 days) in patients with wasp sting-MODS.

Next, ROC curve analysis was used to assess the diagnostic efficacy of CPT1, ACO, GLUT1, IL-6, TNF-*α* and IL-10 on the duration of hospitalization (>2 days). The results showed that CPT1, ACO, GLUT1, IL-6, TNF-α and IL-10 had high diagnostic efficacy for hospitalization duration longer than 2 days, which was mainly demonstrated by CPT1 (AUC = 0.884, 95%CI: 0.838–0.930), ACO (AUC = 0.923, 95%CI: 0.886–0.959), GLUT1 (AUC = 0.947, 95%CI: 0.917–0.976), IL-10 (AUC = 0.931, 95%CI: 0.901–0.975), IL-6 (AUC = 0.927, 95%CI: 0.878–0.975) and TNF-α (AUC = 0.891, 95%CI: 0.835–0.947; Figures 5H,I).

In brief, these results revealed that inflammatory imbalance and metabolic disturbances are independent predictors of prolonged hospitalization in patients with wasp sting-MODS. In addition, CPT1, ACO, GLUT1, IL-6, TNF-α and IL-10 had high diagnostic efficacy in patients with wasp sting-MODS who were hospitalized for more than 2 days.

### 3.6 Analysis of independent risk factors for poor prognosis and survival analysis of wasp sting-MODS patients

**Figure 6:**
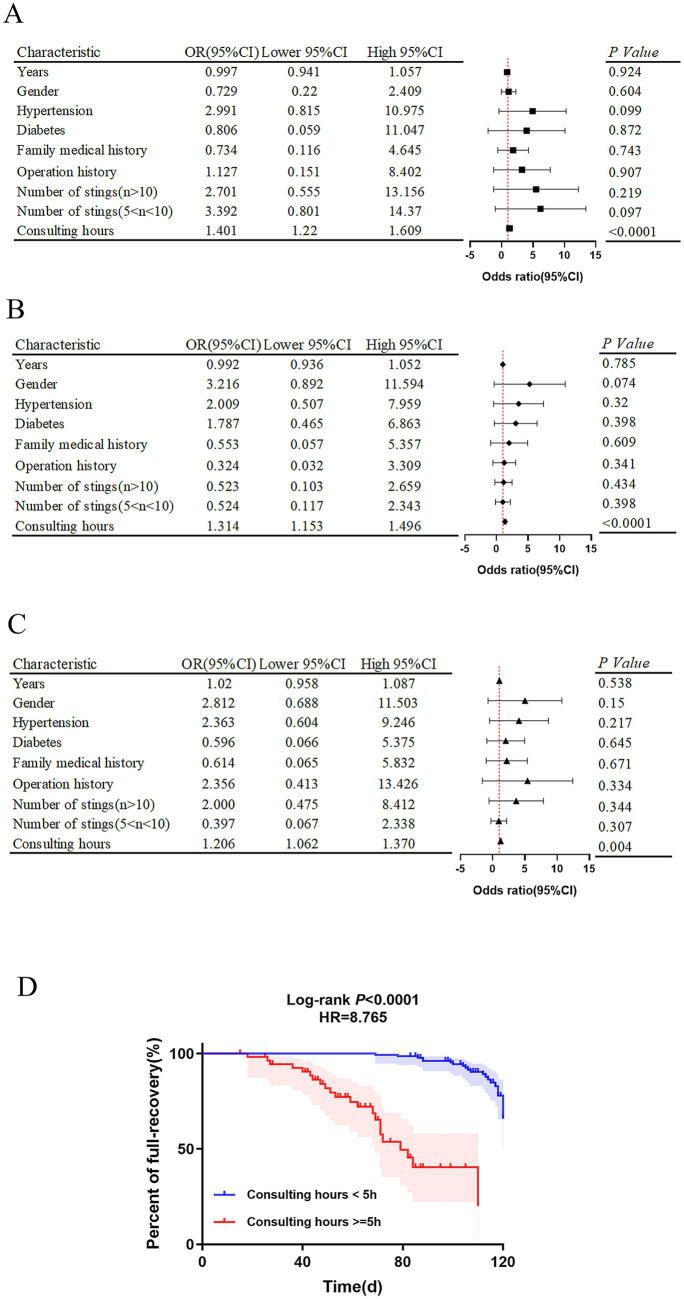
Analysis of independent risk factors for poor prognosis and survival analysis of wasp sting-MODS patients. **(A–C)** Independent risk factors for clinical adverse prognosis in wasp sting-MODS patients analyzed by a multifactorial logistic combined with a random forest approach, mainly including ICU admissions, hemodialysis and death. **(D)** The rate of complete recovery in wasp sting-MODS patients at 120 days after discharge was assessed by survival analysis curves. The results showed that the post-discharge recovery rate for patients with a consultation time < 5 h was 8 times higher than that for patients with a consultation time ≥ 5 h. *p < 0.05, **p < 0.01, ***p < 0.001, ****p < 0.0001, or ns *p* > 0.05.

Primarily, the logistic regression model combined with random forest analysis were developed to evaluate independent risk factors for adverse outcomes in wasp sting-MODS group during hospitalization, with a focus on ICU admission, hemodialysis requirement and mortality. The methodology involved univariate screening of variables, followed by multivariate logistic regression analysis and subsequent integration with random forest modeling to refine risk factor selection. Systemic analysis showed that prolonged consulting time is the only independent predictor with significant positive correlation in three models for predicting in-hospital adverse outcomes in wasp sting-MODS group (Figures 6A–C). The result showed that each additional hour of delayed consultation increases the risk of ICU admission by 40.1%, the need for hemodialysis by 31.4% and the risk of mortality by 20.6% in patients with wasp sting-MODS (Figures 6A–C). Other variables, such as age, gender, hypertension and diabetes did not show a significant effect (Figures 6A–C). Our results confirmed that delayed consultation was significantly associated with higher ICU admission rates (*p* < 0.0001, OR = 1.401, 95%CI: 1.22–1.609), increased need for hemodialysis (*p* < 0.0001, OR = 1.314, 95%CI: 1.153–1.496), and increased in-hospital mortality (*p* < 0.01, OR = 1.206, 95%CI: 1.062–1.370) during hospitalization of patients with wasp sting-MODS (Figures 6A–C).

Post-hoc, survival analysis was further performed to evaluate the predictive value of the independent risk factors for complete recovery within 120 days post-discharge. It revealed that the probability of complete recovery within 120 days post-discharge is 8 times higher (*p* < 0.0001, 95% CI: 2.3–14.5) in patients with a consultation time of <5 h compared to those with a consultation time of ≥5 h (Figure 6D). Thus, this dose-effect relationship implied that early and timely treatment (especially within the golden 5 h) significantly improves the long-term prognosis of patients with wasp sting-MODS, resulting in a higher probability of full recovery up to 4 months after hospital discharge.

## 4 Discussion

This study shows that compared with wasp sting patients who do not develop MODS, wasp sting patients with MODS have significant abnormalities abnormalities in peripheral blood cell count, liver and kidney function, cardiac enzyme profiles, electrolytes and coagulation. These findings are consistent with previous studies including a reported case of AKI, myocardial infarction, stroke and immune thrombocytopenia due to a wasp sting in a 23-year old male in Nepal (19), as well as a study by Liu et al (13) that reported 42 deaths and 1,675 injuries caused by Asian hornets in southern Shaanxi Province, China. The researchers found that during wasp stings, 25.2, 46.6 and 44.7% of the patients developed acute interstitial nephritis, acute toxic hepatitis and acute toxic myocarditis of varying degrees, respectively (13). Of these patients, 99 recovered their renal, hepatic and cardiac functions within 1 month and 4 died after prompt and appropriate treatment, including needle extraction, intravenous methylprednisolone and antihistamines (13). Our findings combined with those of previous studies suggest that multiple organ injury caused by wasp stings can be attributed to three reasons: (1) the direct toxic effects induced by wasp venom components. Wasp venom contains a variety of substances with direct cytotoxicity, including phospholipases A1/A2, hyaluronidase, melittin, and VESCP-M2. These toxins can hydrolyze phospholipids on cell membranes, induce cell swelling and apoptosis, cause cell membrane rupture, destroy cell structure, and impair organ function, subsequently leading to tissue necrosis (20–23). (2) Excessive inflammatory response. The components of wasp venom, acting as “foreign bodies,” activate the body’s immune system. This triggers excessive inflammatory cascade reactions and allergic reactions, which indirectly damage multiple vital organs (24, 25). (3) Microcirculatory dysfunction exacerbates tissue damage. Inflammatory responses and wasp venom damage vascular endothelial cells, activate the coagulation system, and promote microthrombus formation, leading to inadequate tissue perfusion (26). Furthermore, organ injury (such as impaired renal acid excretion and bicarbonate conservation) causes electrolyte disturbances and acid–base imbalances, which further deteriorate cardiac and renal function, ultimately establishing a vicious cycle.

In addition to inducing MODS, the inflammatory response is one of the distinguishing features of wasp sting patients (27). Research has shown that wasp stings result in a strong inflammatory response in the patient, manifested by a systemic hyperinflammatory state and cytokine storm syndrome (28, 29). Our results demonstrated that a significant pro-inflammatory-anti-inflammatory imbalance characterizes wasp sting-MODS patients. This is consistent with Li et al., who demonstrated (30) that cytokine levels were significantly elevated in patients after wasp stings, including IL-2, IL-6, IL-8, IL-10, IL-17, IFN-*γ* and TNF-*α*. Other evidence shows that the levels of these cellular inflammatory factors were positively correlated with white blood cell counts, serum creatinine, and CRP levels, suggesting that the inflammatory response is closely related to the severity of organ damage (30). Furthermore, research has shown that wasp venom stimulates immune cells (macrophages or neutrophils) to massively release inflammatory factors such as TNF-α and IL-6. Excessive release of these factors leads to Systemic Inflammatory Response Syndrome (SIRS), which in turn induces vasodilation, increased permeability, and insufficient tissue perfusion, damaging vital organs including the kidneys and heart (24, 25). Zhang et al. (31) investigated the predictive value of SIRI in the development of MODS in wasp sting patients, and found that SIRI is an independent risk factor for the development of MODS in wasp sting patients, indicating that SIRI can be used as a reliable indicator for the early identification of the occurrence of MODS in patients with wasp stings, and it can help doctors to timely intervention and provide treatment. Mechanistically, components of wasp venom activate the cGAS-STING signaling pathway, which mediates the activation of immune cells and the release of inflammatory mediators, ultimately leading to the release of mitochondrial DNA and an increased inflammatory response (32).

Notably, our study confirmed that lipid metabolism disorders are also a distinctive characteristic of patients with wasp sting-induced MODS. A serum metabolomics study analyzed the dynamics of metabolites in the serum of patients with wasp stings by high performance liquid chromatography–tandem mass spectrometry (33). Researchers identified a total of 838 serum metabolites, revealing that wasp stings induce alterations in distinct metabolic pathways at different time points. Specifically, 289 metabolites exhibited significant differences at 3 h post-sting, with the primary affected pathway being sphingolipid metabolism (33). Certain metabolites exhibited concentration differences across the 3 h, 24 h, and 72 h post-sting groups, with metabolic pathways linked to thiamine metabolism (33). Furthermore, through a retrospective analysis of 212 patients with wasp stings, Quan et al. (34) identified that serum TC, HDL, and LDL in these patients were significantly decreased, and this was associated with the severity of the condition. In contrast, our study found that serum levels of TC, TG, and LDL were significantly increased in patients with wasp sting-MODS, while HDL levels were significantly decreased. For this contradictory conclusion, we provide the following explanations: (1) bidirectional fluctuations in disease progression. We measured lipid levels within 48 h of hospital admission, whereas the cited study assessed them at 24 h. During the early phase following wasp stings (within 24 h), the body undergoes acute stress, potentially accelerating fat mobilization. This leads to rapid consumption of early-stage lipids, resulting in a transient decrease. However, with the persistence of SIRS and intensified oxidative stress at 48 h, hepatic lipid metabolism becomes disrupted, while lipolysis in peripheral tissues may continue to increase. These factors collectively contribute to a reactive elevation in serum lipid levels. (2) Inflammatory cells exhibit heightened energy demands. As previously stated, wasp venom activates numerous inflammatory cells, causing their energy requirements to surge dramatically. When glucose supply becomes insufficient, the body increases lipid breakdown to fuel these cells, ultimately leading to elevated blood lipid levels. (3) The cumulative effects of multiple organ damage. MODS may not yet be fully manifested within 24 h. By 48 h, the liver, as the central organ for lipid metabolism, may have accumulated sufficient functional impairment to significantly diminish its capacity for lipid clearance. Concurrently, secondary renal damage may further compromise lipoprotein excretion, collectively contributing to the secondary elevation of lipid levels observed at the 48 h. (4) Heterogeneity of the study population. Different cohorts exhibit variations in wasp species, sting counts, basal metabolic status, and genetic backgrounds. Following severe wasp sting stress, patients in this study may present with delayed elevations in lipid metabolism disorders, fundamentally differing from groups with distinct baseline states (manifesting as early decreases).

Combined evidence from published investigations and our results collectively indicates that metabolic disorders and inflammatory imbalances in wasp sting-MODS patients do not exist independently of each other, but rather synergistically contribute to multiorgan damage. The pathogenesis of wasp sting-MODS is closely associated with metabolic disturbances (dysglycemia and dyslipidemia) and inflammatory imbalance (hyperactivation of pro-inflammatory cytokines and suppression of anti-inflammatory mediators). Moreover, the gradient alterations in metabolic indices as well as inflammatory markers may serve as potential biomarkers for assessing disease severity and stratifying risks in patients with wasp sting-MODS.

Our results suggested that peripheral blood leukocytes in patients with wasp sting-MODS not only have abnormally increased numbers, but also have elevated gene expression gradients for key enzymes of glucose and lipid metabolism (CPT1, ACO and GLUT1). This alteration may promote the onset of MODS through a cascade reaction involving “enhanced energy supply, amplified inflammatory storm, exacerbated organ damage,” with the specific mechanism as follows: (1) GLUT1-mediated aerobic glycolysis enhancement activates the “rapid energy recharge” mechanism in inflammatory cells. As the key protein for glucose transmembrane transport, elevated GLUT1 expression increases glucose uptake in inflammatory cells (35), driving cellular metabolism toward aerobic glycolysis for rapid energy supply (36). This fulfills the substantial energy demands of inflammatory cell activation during the wasp sting-MODS phase. (2) CPT1 and ACO synergistically activate fatty acid *β*-oxidation. As the wasp sting injury progresses, heightened glucose consumption creates an “energy deficit,” prompting inflammatory cells to initiate the fatty acid β-oxidation pathway by upregulating CPT1 and ACO expression (36). However, the synergistic activation of glycolysis and fatty acid oxidation comes at the cost of lactic acid accumulation, which induces acidification of the local microenvironment and generates substantial amounts of ROS. This leads to mitochondrial dysfunction and inflammasome activation, promoting the release of cytokines such as TNF-*α* and IL-6. Consequently, it exacerbates SIRS, thereby laying the groundwork for the development of MODS (37). This also explains why our subsequent findings indicated that IL-6, TNF-α, IL-10, CPT1, ACO and GLUT1 are independent predictors of prolonged hospitalization (>2 days) in patients with MODS following wasp stings, and exhibit high diagnostic efficacy. Thus, reprogramming of leukocyte metabolism may be an important mechanism driving the synergistic deterioration of metabolic disorders and inflammatory imbalance. This finding provides a new perspective to analyze the pathological process of wasp sting-MODS, and may lead to the exploration of interventional targets for key metabolic enzymes to improve clinical prognosis in the future.

Despite multiple studies focused on observing and analyzing the potential for wasp stings to lead to severe systemic reactions, including MODS, acute respiratory distress syndrome and secondary infections (4, 19, 38), very few have examined the adverse prognosis of wasp sting-MODS patients during hospitalization and survival post-discharge. There is evidence that age and time between sting and medical consultation are risk factors for severe allergic reactions or organ dysfunction or even death (39). Similarly, M. Wang and colleagues demonstrated that female gender, age, number of stings, and intoxication severity score were independent risk factors for death in wasp sting patients (14). However, our study demonstrated that prolonged medical visit duration was the sole independent risk factor associated with adverse outcomes in patients with wasp sting-MODS during hospitalization, which primarily encompassed ICU admission, hemodialysis, and mortality. Other variables, including age, gender, hypertension and diabetes, among others, were not identified as significant risk factors and exhibited no statistical significance. These differences may be related to the sample size and the species of wasps or other factors. It is necessary to further expand the sample size to clarify. Furthermore, our result demonstrated that wasp sting-MODS cases receiving medical intervention within 5 h post-sting exhibited an 8-fold higher probability of achieving complete recovery at 120 days post-discharge compared to those with delayed treatment initiation (>5 h, 95% CI 2.3–14.5). This pronounced time-dependent effect highlights the critical importance of early therapeutic interventions during the 5-h golden window post-envenomation, which significantly optimizes long-term functional outcomes in these patients. Therefore, early standardized medical care plays a crucial role in mitigating persistent organ damage and improving long-term prognosis.

This study has several limitations. First, as a single-center study with a limited sample size, its findings may be affected by selection bias and reduced statistical power; although multivariate analysis was used to minimize confounding factors, the generalizability of the results still needs validation via large-scale, multicenter prospective studies. Second, incomplete clinical documentation excluded critical variables such as patients’ pre-existing comorbidities factors (immunocompromising conditions or immunosuppressive agent use) that could significantly impact infection susceptibility and inflammatory response dynamics, and prevented full control for “unrecorded medication use” (over-the-counter antihistamines taken before admission but unreported), which may have slightly compromised the accuracy of inflammatory or metabolic indicator analysis. Third, only common confounding factors were adjusted for, with no inclusion of rare ones (genetic polymorphisms related to toxin metabolism), limiting the results’ generalizability to populations with specific genetic backgrounds. Finally, the current 4-month survival analysis lacks long-term outcome data; subsequent multicenter prospective studies will extend follow-up to evaluate 1-year and 3-year survival outcomes, thereby strengthening the robustness of prognostic stratification and risk–benefit assessment for post-discharge interventions.

## 5 Conclusion

In summary, this study systematically demonstrated that wasp sting-MODS exhibits multi-system abnormalities, with disrupted expression of key enzymes in leukocyte glycolipid metabolism closely associated with serum glycolipid abnormalities. Findings indicated that IL-6, TNF-*α*, IL-10, CPT1, ACO, and GLUT1 serve as independent predictors of prolonged hospitalization with significant diagnostic efficacy. Crucially, delayed medical consultation is the only independent risk factor, and early intervention within 5 h post-wasp sting substantially increases the probability of complete functional recovery at 4 months. Therefore, future research should further enhance preventive measures and increase clinicians’ awareness of wasp stings-MODS, as well as develop new biomarkers and predictive models to provide clinicians with a more accurate basis for diagnosis and treatment, thereby improving patient prognosis.

## Data Availability

The raw data supporting the conclusions of this article will be made available by the authors, without undue reservation.
