# Supplementary material for: Multisystem injury after wasp stings in the Qinling range, Shaanxi, China: clinical profile and independent predictors of poor outcome
Source: Front Med (Lausanne). 2025 Nov 20;12:1716614. doi: 10.3389/fmed.2025.1716614 (PMC12675340; doi:10.3389/fmed.2025.1716614)
Supplement: Supplementary file 1 [file Table_1.docx]

**Supplementary table 1.** Comparison of Laboratory data of controls, wasp sting-NMODS and wasp sting-MODS group

| Laboratory data | Control  (n=147) | Wasp Sting-NMODS  (n=132) | Wasp Sting-MODS  (n=55) | *P Value* |
| --- | --- | --- | --- | --- |
| **Peripheral Blood** |  |  |  |  |
| NEU(x10^9^/L) | 3.18(2.39, 4.03) | 8.44(4.72, 12.44) | 13.88(10.44, 18.12) | <0.0001 |
| LYM(x10^9^/L) | 1.93(1.06, 2.73) | 1.36(0.78, 2.20) | 0.95(0.60, 1.88) | <0.0001 |
| MON(x10^9^/L) | 0.40(0.31, 0.52) | 0.48(0.31, 0.64) | 0.62(0.47, 0.87) | <0.0001 |
| EOS(x10^9^/L) | 0.10(0.05, 0.15) | 0.03(0.01, 0.09) | 0.20(0.00, 0.40) | <0.0001 |
| BAS(x10^9^/L) | 0.03(0.02, 0.05) | 0.02(0.01, 0.04) | 0.02(0.01, 0.04) | 0.001 |
| HCT(%) | 44.17±4.09 | 41.46±6.18 | 35.19±8.03 | <0.0001 |
| MCV(fl) | 92.72±4.92 | 92.15±8.21 | 94.03±5.83 | 0.200 |
| MCH(pg) | 30.36±1.94 | 31.15±2.47 | 31.85±2.05 | <0.0001 |
| MCHC(g/L) | 327.43±11.90 | 333.81±31.68 | 338.43±18.78 | 0.004 |
| RDW | 12.65±1.08 | 12.88±2.67 | 13.27±1.42 | 0.109 |
| MPV(fl) | 10.76±0.93 | 10.36±1.08 | 11.17±1.53 | <0.0001 |
| PDW | 12.88±2.15 | 11.90±2.85 | 13.27±1.42 | <0.0001 |
| PCT(%) | 0.24±0.06 | 0.24±0.07 | 0.22±0.09 | 0.062 |
| **Liver** |  |  |  |  |
| ALB(g/L) | 43.69±2.76 | 43.63±6.69 | 30.67±7.22 | <0.0001 |
| AST(U/L) | 24.02±8.35 | 31.67±12.77 | 532.75±184.19 | <0.0001 |
| PA(mg/L) | 303.59±56.97 | 261.30±63.04 | 125.73±41.04 | <0.0001 |
| **Kidney** |  |  |  |  |
| UA(μmol/L) | 347.41±99.93 | 326.41±69.31 | 562.42±56.45 | <0.0001 |
| **Myocardium** |  |  |  |  |
| HBDH(U/L) | 132.0(112.0, 153.0) | 153.5(114.3, 180.8) | 1094.0(703.5, 1529.0) | <0.0001 |
| **Electrolyte** |  |  |  |  |
| Mg(mmol/L) | 1.09±0.19 | 0.89±0.14 | 0.88±0.22 | <0.0001 |
| P(mmol/L) | 1.28±0.23 | 1.11±0.24 | 0.77±0.17 | <0.0001 |

NEU: Neutrophils, LYM: Lymphocyte, MON: Monocytes, EOS: Eosinophils, BAS: Basophils, HCT: Hematokrit, MCV: Mean corpuscular volume, MCH: Mean corpuscular hemoglobin, MCHC: Mean corpuscular-hemoglobin concentration, RDW: Red blood cell distribution width, MPV: Mean platelet volume, PDW: Platelet distribution width, PCT: Thrombocytocrit, ALB: Albumin, AST: Glutamic oxaloacetic transaminase, PA: Prealbumin, UA: Uric acid, Mg: Magnesium, P: Phosphorus, HBDH: Hydroxybutyrate dehydrogenase.
